# Pollinator Competition as a Driver of Floral Divergence: An Experimental Test

**DOI:** 10.1371/journal.pone.0146431

**Published:** 2016-01-27

**Authors:** Ethan J. Temeles, Julia T. Newman, Jennifer H. Newman, Se Yeon Cho, Alexandra R. Mazzotta, W. John Kress

**Affiliations:** 1 Department of Biology, Amherst College, Amherst, Massachusetts, United States of America; 2 Department of Botany, National Museum of Natural History, MRC-166, Smithsonian Institution, Washington, District of Columbia, United States of America; Central China Normal University, CHINA

## Abstract

Optimal foraging models of floral divergence predict that competition between two different types of pollinators will result in partitioning, increased assortative mating, and divergence of two floral phenotypes. We tested these predictions in a tropical plant-pollinator system using sexes of purple-throated carib hummingbirds (*Anthracothorax jugularis*) as the pollinators, red and yellow inflorescence morphs of *Heliconia caribaea* as the plants, and fluorescent dyes as pollen analogs in an enclosed outdoor garden. When foraging alone, males exhibited a significant preference for the yellow morph of *H*. *caribaea*, whereas females exhibited no preference. In competition, males maintained their preference for the yellow morph and through aggression caused females to over-visit the red morph, resulting in resource partitioning. Competition significantly increased within-morph dye transfer (assortative mating) relative to non-competitive environments. Competition and partitioning of color morphs by sexes of purple-throated caribs also resulted in selection for floral divergence as measured by dye deposition on stigmas. Red and yellow morphs did not differ significantly in dye deposition in the competition trials, but differences in dye deposition and preferences for morphs when sexes of purple-throated caribs foraged alone implied fixation of one or the other color morph in the absence of competition. Competition also resulted in selection for divergence in corolla length, with the red morph experiencing directional selection for longer corollas and the yellow morph experiencing stabilizing selection on corolla length. Our results thus support predictions of foraging models of floral divergence and indicate that pollinator competition is a viable mechanism for divergence in floral traits of plants.

## Introduction

Competition is considered to be a major force driving natural selection within and between species [1, 2, 3]. In pollination systems, the role of competition is unusual in that pollinators may compete for plants as much as plants compete for pollinators [4]. Theoretical work suggests that both exploitative and interference competition between pollinators can contribute to floral divergence [5, 6, 7, 8, 9, 10]. The basis of these foraging models is that (i) variability in floral traits leads to resource partitioning which results from competitive asymmetries when pollinators compete for resources; (ii) resource partitioning by pollinators increases assortative mating within floral phenotypes, and (iii) resource partitioning and assortative mating lead to floral divergence [10]. These models have been used to explain divergence in floral coloration and the evolution of long floral tubes [7, 8, 9, 11]. Despite numerous examples of competition for nectar resources among pollinators from various taxa (e.g., birds, bees, bats; [12, 13, 14, 15, 16, 17, 18, 19, 20]), the link between pollinator competition and floral divergence has been assumed rather than established [21].

**Fig 1:**
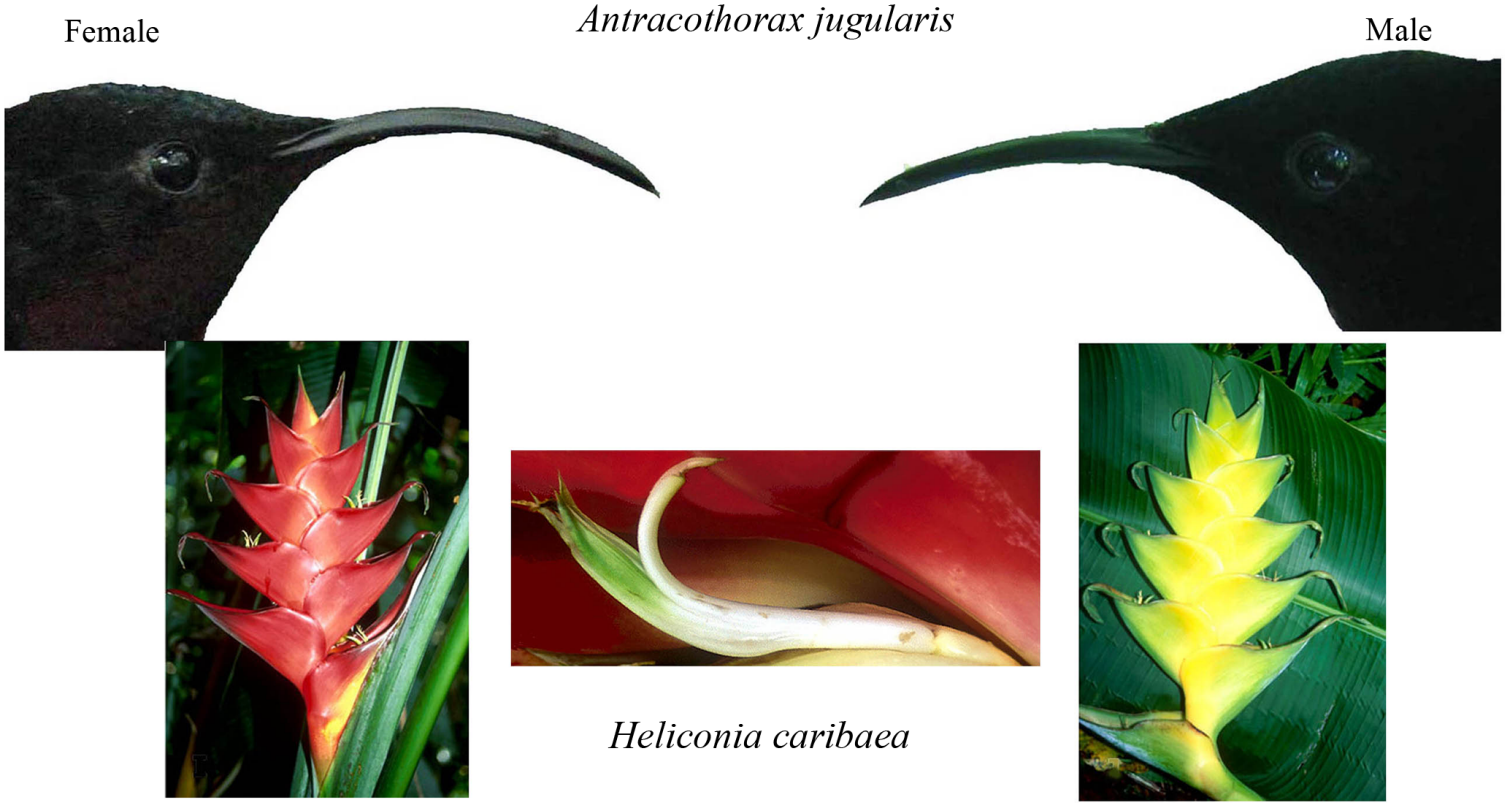
Bills of female and male purple-throated caribs, *Antracothorax jugularis*, inflorescences of the red and yellow morphs of *Heliconia caribaea*, and a representative flower of *H*. *caribaea*.

We present an experimental study of the role of competition in floral divergence using sexes of purple-throated carib hummingbirds (*Anthracothorax jugularis;* formerly *Eulampis*; [22]) as the pollinators and color morphs of *Heliconia caribaea* as the plants. Purple-throated caribs are native to the mountainous islands of the Eastern Caribbean, where their primary food plants are *Heliconia caribaea* and *H*. *bihai*, the only heliconias native to the Lesser Antilles. Male purple-throated caribs are 25% more massive than females, yet females have bills that are 30% longer (Fig 1; [19]). Our previous work [23, 24, 25] demonstrated that males are the primary pollinator of *H*. *caribaea*, which has many bracts (and hence flowers) per inflorescence (6–24) and short flowers (34–40 mm) corresponding to their short bills, larger size, and higher energy requirements. Females are the primary pollinator of *H*. *bihai*, which has fewer bracts per inflorescence (2–8) with long flowers (44–52 mm) corresponding to their long bills, smaller size, and lower energy requirements relative to males. These differences in the use of the two heliconias are associated with asymmetries in competitive abilities: males defend territories of *H*. *caribaea* and their larger size is associated with increased fighting ability relative to females [26]. In contrast, females forage by traplining at undefended plants of the two heliconias and by intruding onto male territories [19, 27], and their longer bills are associated with increased foraging efficiency at long flowers [28]. Furthermore, indirect support for the hypothesis of coevolution between purple-throated caribs and their *Heliconia* food plants is provided by a frequency-dependent polymorphism in bract color, corolla (flower) length, and bract numbers within the two species of *Heliconia* in some but not all populations. In this case floral traits and energy rewards of one morph correspond more closely to the bills, size, and energy needs of males, whereas floral traits and energy rewards of the other morph correspond more closely to the bills, size, and energy needs of females [23, 24]. In these populations, each sex visits the *Heliconia* morph corresponding to its morphology [23, 24, 29].

In an enclosed *Heliconia* garden on the island of Dominica we capitalized on the floral color dimorphism of *Heliconia caribaea* (Fig 1) to conduct an experiment on whether pollinator competition can select for floral divergence. First, we examined whether sexes of purple-throated caribs differed in their use of color morphs of *H*. *caribaea* when foraging alone and whether their use of color morphs changed in competition. Second, using powdered dyes as pollen analogs, we compared within-morph dye transfer (assortative mating) when sexes foraged alone versus when sexes foraged in competition. Lastly, using powdered dyes as pollen analogs, we measured relative fitness of color morphs and natural selection on corolla (flower) length when sexes foraged alone versus when sexes foraged in competition to examine whether competition could lead to floral divergence in bract color or corolla length.

## Methods

Our study was conducted on the property and with the permission of Mr. Mervin Thomas, Warner Rd., Dominica, West Indies (15°23’54”N, 61°23’21”W) from 18 May to 26 June 2012, 16 May to 27 June 2013, and 16 May to 26 June 2015.

### The birds

Individuals of *Anthracothorax jugularis* were captured in the surrounding rain forest using mist-nets and then housed individually in 4 x 3 x 2.2 m tents at the rain forest edge. Natural perches were provided and birds were maintained on a diet of 25% sucrose solution by volume and insects. To prevent re-use in the experiments, birds were fitted with unique color bands to distinguish individuals. Birds were given 24 h in which to acclimate to captivity before use in the experiments. Following the experiments, birds were released to the wild at the capture site. Although we did not attempt to locate birds after their release, sightings of released, color-banded birds during the same and subsequent study years suggests that post-release survival was good. We studied five male-female pairs in 2012, four in 2013, and three in 2015. Our protocol for experiments and care of hummingbirds was approved by the IACUC committee of Amherst College (Animal Welfare Assurance Number 3925–01) and by Jacqueline Andre and Stephen Durand of the Forestry, Wildlife and Parks Division of the Ministry of Agriculture and Forestry of the Commonwealth of Dominica.

### The plants

*Heliconia caribaea* is one of two *Heliconia* species native to the island of Dominica and occurs along roads, trails, rivers, and in forest light gaps from approximately 70 m to 600 m elevation in primary and secondary rainforest. Plants are perennial large herbs and tree-like, with mature plants ranging from 2 to 10 m in height. *Heliconia caribaea* has rhizomatous growth, a musoid growth habit, and produces multiple inflorescences (1–35) that produce flowers over a period of 1–3 months [30]. Inflorescences are formed by large, colorful bracts (modified leaves) and on Dominica, *H*. *caribaea* has two inflorescence color morphs, one with red bracts and the other with yellow bracts (Fig 1; [24]). In *H*. *caribaea*, each bract holds a range of 1–30 flowers over the season, but no more than one flower is produced daily within a bract; anthesis lasts one day [19]. The flowers on *H*. *caribaea* are bisexual, zygomorphic, tubular, and greenish-white in color (Fig 1; [24]). On Dominica, *H*. *caribaea* is visited by both female and especially male purple-throated caribs, with the latter defending clumps of the plants as territories [24, 25, 26]. Green-throated caribs (*A*. *holosericeus*) and Antillean crested hummingbirds (*Orthorhyncus cristatus*) are rare visitors to *H*. *caribaea*, accounting for less than 5% of visits in our 10 years of work on Dominica [24, 25, 26]. Our past work indicates that sexes of purple-throated caribs differentially visit color morphs of *H*. *caribaea* in some but not all populations [24, 29]. It is unclear whether such differential visitation results from differences in preferences or competition.

Pollen grains do not differ in appearance between red and yellow color morphs of *H*. *caribaea*, and as a consequence, we used non-toxic fluorescent powder to assess within-morph dye/pollen transfer (assortative mating) and natural selection on flower length, a common approach in pollination studies [31]. Previous work indicates that *H*. *caribaea* is pollen-limited and thus pollen deposition is a suitable fitness measure [32]. We used Risk Reactor short-wave ultra-violet dyes in blue, yellow, and green. These pigments are off-white under visible light but fluoresce a specific color under short-wave ultra-violet light (< 365 nm). We specifically chose short-wave dyes because hummingbird UV-sensitivity is in the long-wave spectrum [33]. Particle size (3–6 μm) is similar to that used in other dye experiments [31]. We verified that dye was an appropriate analog for pollen by conducting pollen-carryover trials using 10 different birds. Birds were first offered a newly-dehisced *H*. *caribaea* flower to which dye had been liberally applied with a toothpick, and then were allowed to visit four to six flowers in succession that had been emasculated by removing their anthers (90% of purple-throated carib foraging bouts consist of five or fewer flowers). The number of dye particles received per flower was a significant predictor of the number of pollen grains received per flower (*R*^*2*^ = 0.9, *P* < 0.001, *N* = 54, S1 Dataset). Similarly, the slopes and intercepts of the regressions of dye/pollen received versus flower order did not differ significantly, nor did the number of pollen or dye grains received for flowers of a given order (*P*’s > 0.1, ANCOVA and *t*-tests, respectively; see S1 Fig and S1 Text for detailed statistics). Thus, powdered dyes are a good proxy for pollen in *H*. *caribaea*.

### The experiments

**Fig 2:**
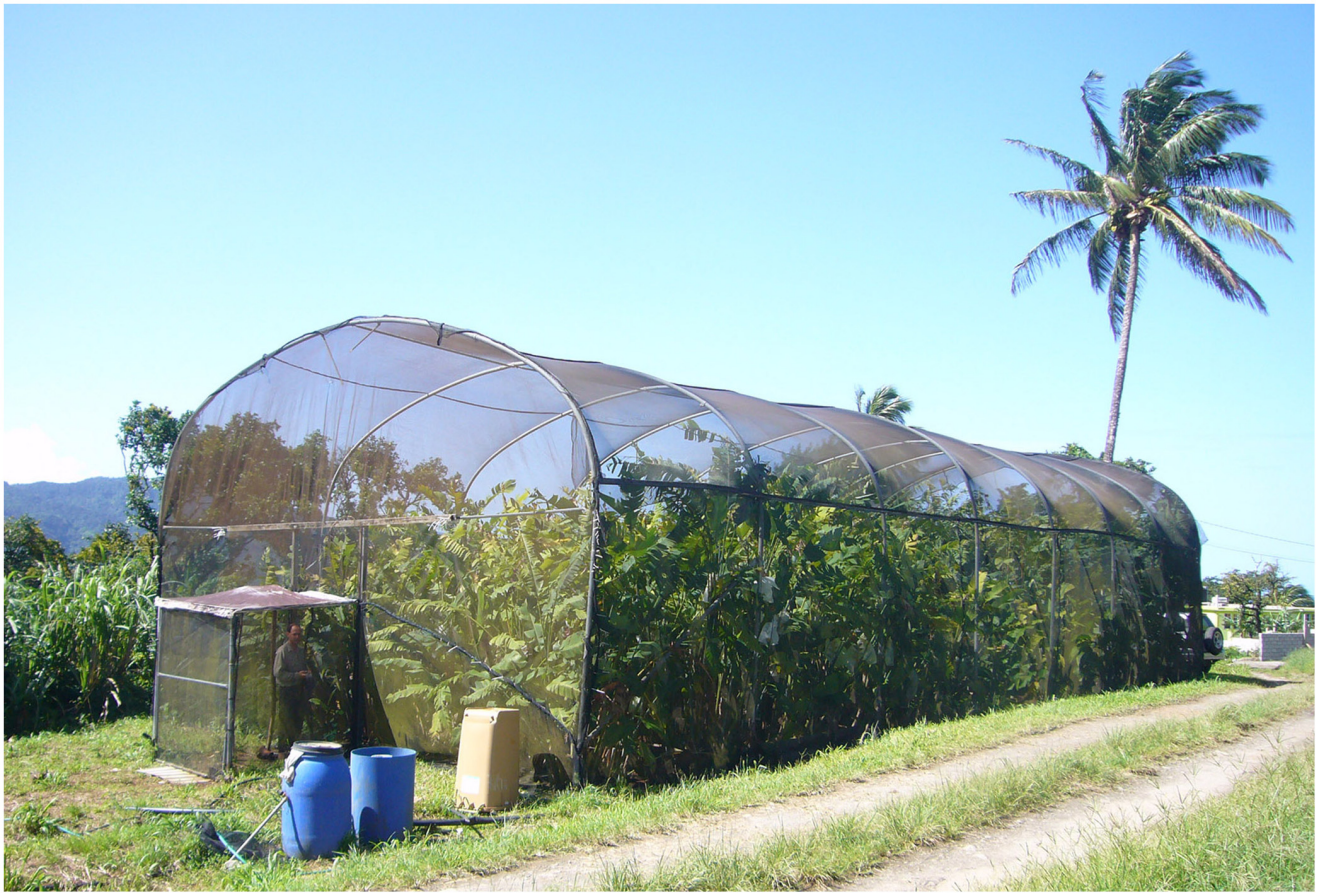
Enclosed *Heliconia* garden on Dominica. The dimensions of the screenhouse are 30 m x 10 m x 7 m.

We conducted experiments in an enclosed *Heliconia* garden (Fig 2). Plants were collected at various sites in 2005 and have been maintained in a common garden since then; we chose individual plants to insure a broad range in flower length. The garden is contained within an enclosed shadehouse measuring 30 m L x 10 m W x 7 m H, approximately the size of a basketball court (Fig 2). *Heliconia caribaea* territories of male purple-throated caribs are roughly 100 m^2^ [19, 26]; thus, the size of the garden is approximately three male territories. We know from banding studies [34] that males and females will remain faithful to the same patch of heliconias for as long as five years; males have been observed inspecting their *H*. *caribaea* patches even in months when the patches are not in flower [34]. Such site fidelity is common in non-migratory tropical birds [35, 36]. We thus feel that our garden mimics a spatial scale appropriate for this tropical hummingbird species.

To determine whether competition between male and female purple-throated caribs could result in the partitioning and floral divergence of the red and yellow color morphs of *H*. *caribaea*, we needed to establish whether male and female purple-throated caribs preferred one color morph as opposed to the other and patterns of pollination in relation to color morph and floral traits. These measurements provided a frame of reference for how each sex behaved in the absence of competition and allowed us to examine whether and how competition altered preferences and pollination patterns. For the preference experiment, a single purple-throated carib was introduced into the garden and given approximately 20 h to acclimate to 6 plants of the red morph of *H*. *caribaea* and 6 plants of the yellow morph of *H*. *caribaea*; inflorescences of the other plants in the garden were covered with opaque plastic bags to prevent feeding visits. The competition experiment was similar except that we introduced a male and female pair of purple-throated caribs into the garden. In each experimental trial, a male and female were matched so that they visited the same array of 6 red and 6 yellow plants alone and the same array of plants in competition. We manipulated plants to ensure equal numbers of inflorescences (10 per plant) and flowers (10 per plant).

Based upon measurements from our garden, *H*. *caribaea* produced approximately 100 μl of nectar per flower per day with a concentration of approximately 22% sucrose (see S1 Table); thus a single flower produced 0.946 cal./ μl of nectar. The number of flowers (120) on the 12 plants used in each experimental trial was approximately 1.12x a male’s daily energy requirements of 10.7 Kcal and 1.26x a female's daily energy requirements of 9.5 Kcal (see [19, 26] for details on foraging energetics). Consequently, when foraging alone, each male or female had sufficient nectar to meet their energy requirements, but when foraging together, nectar rewards were below their daily energy requirements, thus ensuring competition for a limiting resource. We selected our 12 plants for each experimental trial from 14 plants in 2012 (7 red, 7 yellow), 16 plants in 2013 (7 red, 9 yellow), and 15 plants in 2015 (8 red, 9 yellow). In each year of the study, neither nectar volume, nectar concentration, number of bracts per inflorescence, nor corolla length differed significantly between red and yellow color morphs (*P* > 0.1, *t*-tests, see S1 Table).

Following the acclimation period and prior to the start of behavioral observations, anthers on half of the ten flowers on each of the 12 plants were dyed with non-toxic fluorescent powder (different colors were used for each *Heliconia* morph and were rotated between trials). Bracts were marked using Sharpie markers so that we could distinguish dyed from undyed flowers at the end of the experiment. We then watched each bird or pair for five hours (from approximately 9:00 AM to 2:00 PM), during which time we recorded the sex of the visitor, the exact time a bird visited a plant, the morph of the plant and its identification number on each visit, and the number of inflorescences and flowers visited on each visit. In order to distinguish visited from unvisited flowers, we marked inflorescences by tying numbered flagging tape to their shoots following initial visits. We also recorded the number of chases of females by males during the competition trials.

Following observations, we collected visited, undyed flowers and examined them under a 40X microscope with UV lighting for dye particles deposited on their stigmas. Because the color of bracts changes in hue, brightness, and intensity over the lifetime of the inflorescence, we did not estimate selection on color as a continuous trait within each morph. Rather, we treated color as a binary trait (red, yellow) and compared dye deposition between color morphs. In addition, we estimated selection on corolla length, and measured the corolla length (in mm) of each flower that received dye using digital calipers. Given the difference in bill length between male and female purple-throated caribs (Fig 1), we expected that sexes might differ in the direction and magnitude of selection on corolla length, with females driving selection for longer corollas and males driving selection for shorter corollas.

### Statistical analyses

The unit of replication in our experiments was male-female pairs. The large size of *H*. *caribaea* plants relative to the size of our enclosure, as well as the timing of flowering of plants, necessitated that we reuse some of the plants during sequential experimental trials (median = 7 plants, range = 6–12). As a consequence, apparent preference for a color morph of *H*. *caribaea* could result from preference for particular plants and not color morphs. To reduce this possibility, we conducted Monte Carlo exact tests of independence with 10^6^ draws to compute *P*-values using PROC FREQ in SAS Version 9.3 (SAS Institute, Cary, NC, USA) between individuals of a sex that exhibited a preference for a color morph, which in this case was males for yellow, grouping unshared yellow plants in the category “Unshared”. Given our sample size of 12 pairs, there were 66 comparisons between males for both the no-competition and competition trials. We judged a Bonferroni adjustment of *P*-values (0.05/66 = 0.00076) as too conservative in that it would eliminate many pairs for which differences were highly significant. We thus used a Benjamini and Yekutieli threshold *P*-value of 0.01037 for multiple tests; this adjustment of *P*-values is considered to be a compromise between the less-conservative Benjamini and Hochberg adjustment and the highly conservative Bonferroni adjustment [37]. We then eliminated one of the male-female pairs in which males did not differ significantly from each other in their visits to plants of the yellow morph unless 50% or more of plants were unshared. This culling criterion reduced the data set from 12 pairs to 8 pairs.

We used the proportion of visits to the yellow morph of *H*. *caribaea* for each bird as a measure of preference and tested the significance of preference for males and females alone and in the competition trials against a null hypothesis of no preference (*i*.*e*., 50% of visits were to the yellow morph; S2 Dataset) using one-sample *t*-tests in Minitab Release 14 (Minitab Inc., State College, PA, USA). We compared preferences of males and females when foraging alone and in competition using paired *t*-tests in Minitab with sequential Bonferroni adjustments of *P*-values [38]; data were arcsine-transformed prior to analysis in order to meet assumptions of parametric tests. To assess assortative mating, we used paired *t*-tests in Minitab with sequential Bonferroni adjustments and arcsine-transformations of the data to compare the proportion of within-morph pollination events for males and females foraging alone and for both sexes combined when foraging in competition (note that because males and females sometimes visited the same plants in competition trials, we could not reliably assign pollination to separate sexes; see S3 Dataset).

We used the average number of dye particles received per plant as our measure of fitness and calculated this measure by first taking the average number of dye particles received per plant in each of the eight trials for males alone, females alone, and males and females in competition. We then took the average of those averages to obtain the average number of dye particles received per plant for males alone, females alone, and males and females in competition (S4 Dataset). To assess selection on color morphs, we compared the average number of dye particles received per plant for the red and yellow morphs using *t*-tests; separate tests were conducted for the male, female, and competition trials. We estimated selection on corolla length using standard selection analysis [39, 40], treating the red and yellow color morphs separately. Prior to analysis we standardized corolla length (*z*_*i*_) to a mean equal to zero and to a variance equal to one. Our fitness measure, mean dye particles received per plant, was converted to relative fitness (*w*_*i*_) by dividing individual fitness by mean fitness [39]. Fitness measures were relativized and corolla lengths were standardized separately for each of the three experimental trials (females alone, males alone, and males and females in competition).

We estimated net selection (direct and indirect selection resulting from phenotypically correlated traits) on a corolla length using univariate regression models. Directional selection was estimated by the linear model (*w′*_*i*_ *= c + β*_*uni*_ *z*_*i*_) whereas stabilizing or disruptive selection was estimated by the quadratic model (*w′*_*i*_ *= c + β′*_*I*_ *z*_*i*_ *+ β*_*2*_ *z*^*2*^_*i*_), where (*γ*_*i*_ = 2 *β*_*2*_) is the univariate nonlinear selection gradient. In these analyses, the univariate linear selection gradient is equivalent to the standardized selection differential [41, 42]. Because we predicted that direction of selection on corolla length should differ between the sexes, with short-billed males selecting for shorter corollas and long-billed females selecting for longer corollas, we first fitted univariate linear regression models and then fitted univariate nonlinear models. We then compared the fit of the linear and non-linear models by calculating the *P-*value of the increase in *R*^*2*^ [43].

## Results

### Pollinator visits

**Fig 3:**
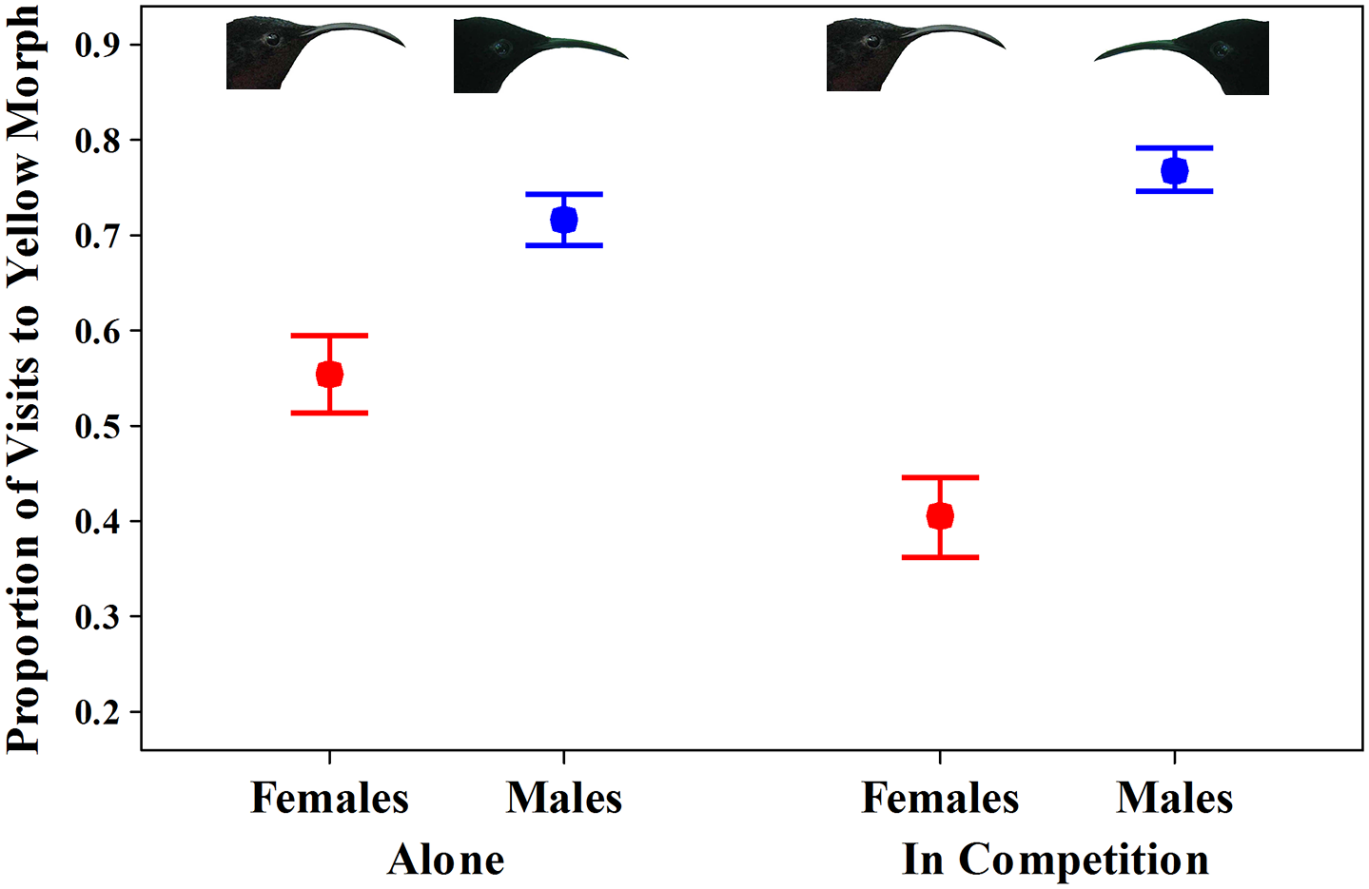
Proportion of visits (Mean ± SE) to the yellow morph of *Heliconia caribaea* by eight male (blue) and eight female (red) purple-throated caribs when foraging alone and in competition. Differences between males and females are significant for the competition trials as are differences between females when foraging alone and in competition. See text for statistics.

When foraging alone, 71% ± 3% of male visits were to the yellow morph of *H*. *caribaea*, and male preference for the yellow morph of *H*. *caribaea* deviated significantly from the null expectation of 50% (*t* = 7.81, *P* < 0.001, *N* = 8 males, 427 visits; Fig 3). In contrast, when foraging alone, 55% ± 12% of female visits were to the yellow morph of *H*. *caribaea*, which was not significantly different from the null expectation of 50% (*t* = 1.35, *P* = 0.22, *N* = 8 females, 283 visits; Fig 3). The difference between male and female purple-throated caribs in the proportion of their visits to the yellow *H*. *caribaea* color morph was not significant under a Bonferroni sequential adjustment, although the individual test was significant (*t* = 2.60, *P* = 0.036, *N* = 8 pairs).

When in competition with females, males continued to exhibit a preference for the yellow morph of *H*. *caribaea*, with 77% ± 3% of their visits to that morph, significantly greater than null expectations of 50% (*t* = 11.43, *P* < 0.001, *N* = 8 males, 406 visits; Fig 3). The proportion of male visits to the yellow morph when in competition did not differ significantly from the proportion when foraging alone (*t* = 1.66, *P* = 0.14, *N* = 8 males). In contrast, when in competition with males, females shifted to foraging more frequently at the red morph of *H*. *caribaea*, and only 40% ± 11% of their visits were to the yellow morph of *H*. *caribaea*, significantly less than null expectations of 50% (*t* = 2.35, *P* = 0.05, *N* = 8 females, 325 visits; Fig 3). The proportion of female visits to the yellow morph when in competition (40%) was significantly less than proportion when foraging alone (55%; *t* = 4.00, *P* = 0.005, *N* = 8 females). Differences between male and female purple-throated caribs in the proportion of their visits to the yellow *H*. *caribaea* color morph when in competition were significant (*t* = 5.87, *P* < 0.001, *N* = 8 pairs).

The change in female visitation behavior from no significant difference in use of color morphs when foraging alone to significant over-visitation of the red morph when in competition was likely the result of direct aggression in which males competitively excluded females from the yellow morph of *H*. *caribaea*. This competitive exclusion consisted of the male chasing the female an average of 21 ± 5 times per experimental trial (*N* = 8 trials) when they attempted to feed at plants of the yellow morph as well as males “guarding” plants of the yellow morph by perching in or next to them, similar to their behavior on natural territories [19, 26]. Females were not observed chasing males in any trials nor were they seen feeding from the same plant at the same time as males.

### Assortative mating in plants

**Fig 4:**
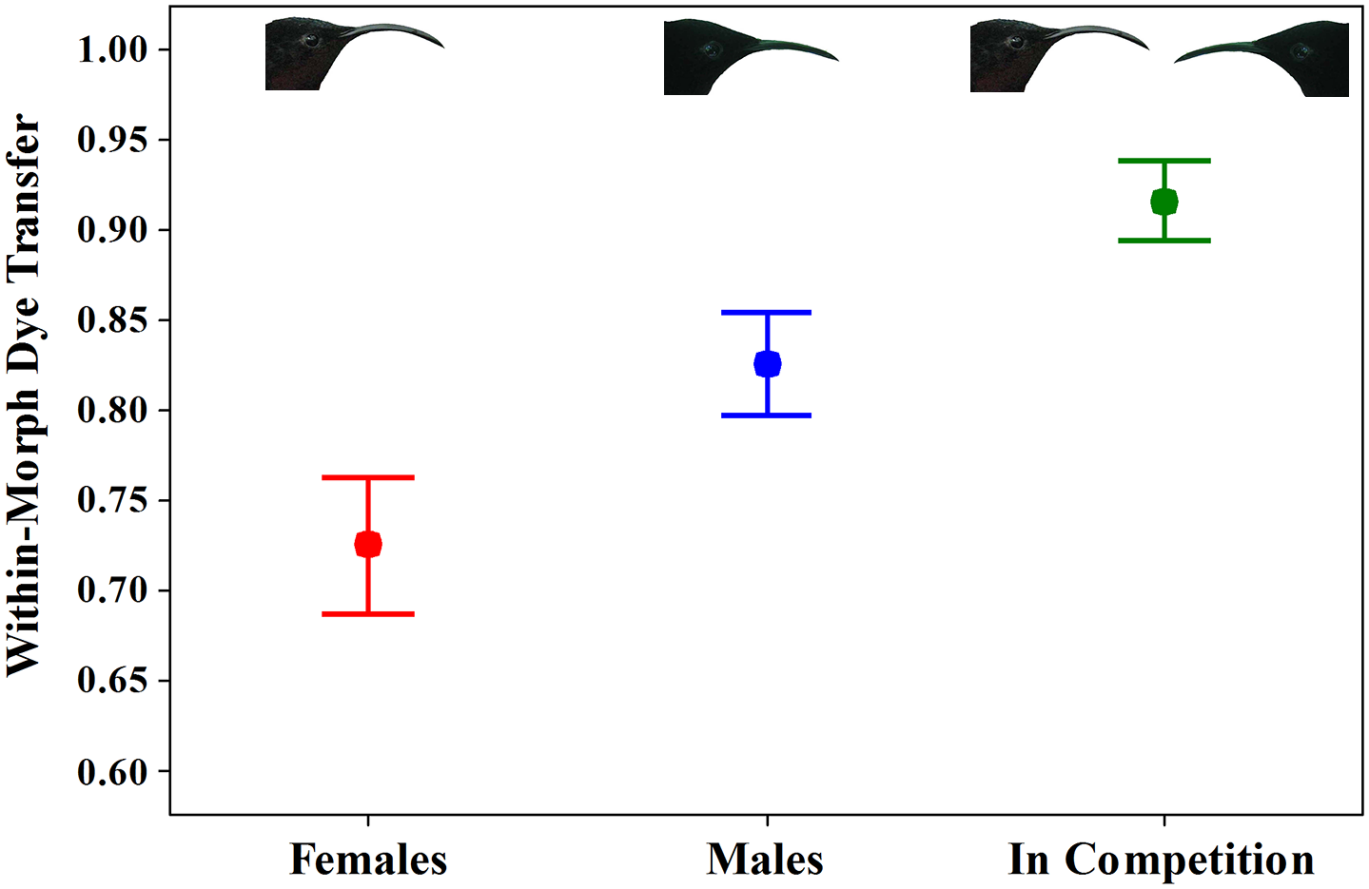
Proportion of dye transferred within color morphs of *Heliconia caribaea* for eight male (blue) and eight female (red) purple-throated caribs when foraging alone and in competition (green). Because males and females sometimes visited the same plants in competition trials, we could not reliably assign pollination to separate sexes. The difference between females when foraging alone and the competition trial is significant. See text for statistics.

When foraging alone, 82% ± 3% of dye transfers by males and 72% ± 4% of dye transfers by females were between plants of the same color morph; the difference between males and females was not significant with a Bonferroni sequential adjustment although the *P-*value for the individual test was significant (*t* = 2.35, *P* = 0.05, *N* = 8 pairs, 319 flowers receiving dye; Fig 4). Competition increased assortative mating and 92% ± 2% of dye transfers were between plants of the same color morph (Fig 4; *N* = 8 pairs, 226 flowers receiving dye). Assortative mating under competition differed significantly from assortative mating when females were foraging alone but not for males (competition versus females: *t* = 6.92, *P* < 0.001, *N* = 8; competition versus males: *t* = 1.72, *P* = 0.13, *N* = 8).

### Selection on bract color

**Fig 5:**
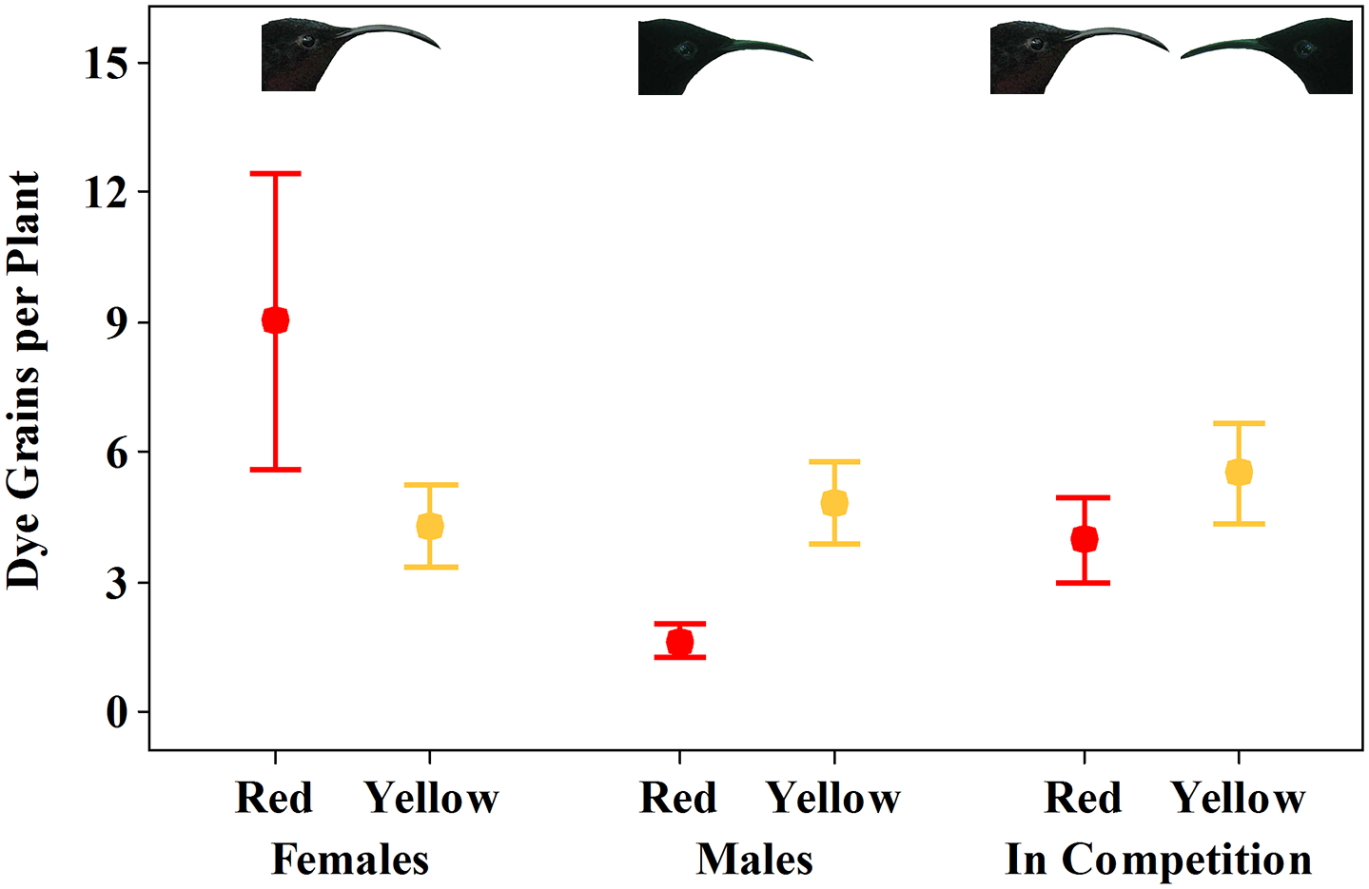
Dye grains received (Mean ± SE) per plant of red and yellow morphs of *H*. *caribaea* for eight male and eight female purple-throated caribs when foraging alone and in competition. See text for statistics.

Males deposited significantly more dye particles per plant for the yellow morph of *H*. *caribaea* than for the red morph of *H*. *caribaea* (Fig 5; *t* = -3.09, *P* = 0.01, *N =* 9 red plants, 86 flowers and *N* = 10 yellow plants, 138 flowers; S4 Dataset). Females, on the other hand, deposited more dye particles per plant for the red morph of *H*. *caribaea* as opposed to the yellow morph of *H*. *caribaea*, although this difference was not significant and variance in dye deposition among plants of the red morph was high (Fig 5; *t* = 1.33, *P* = 0.21, *N* = 9 red plants, 85 flowers and *N* = 9 yellow plants, 104 flowers). In contrast, in competition, dye deposition per plant was similar for the red and yellow morphs and the difference between morphs was not significant (Fig 5; *t* = 1.32, *P* = 0.32, *N =* 10 red plants, 152 flowers and 10 yellow plants, 199 flowers).

### Selection on corolla length

**Fig 6:**
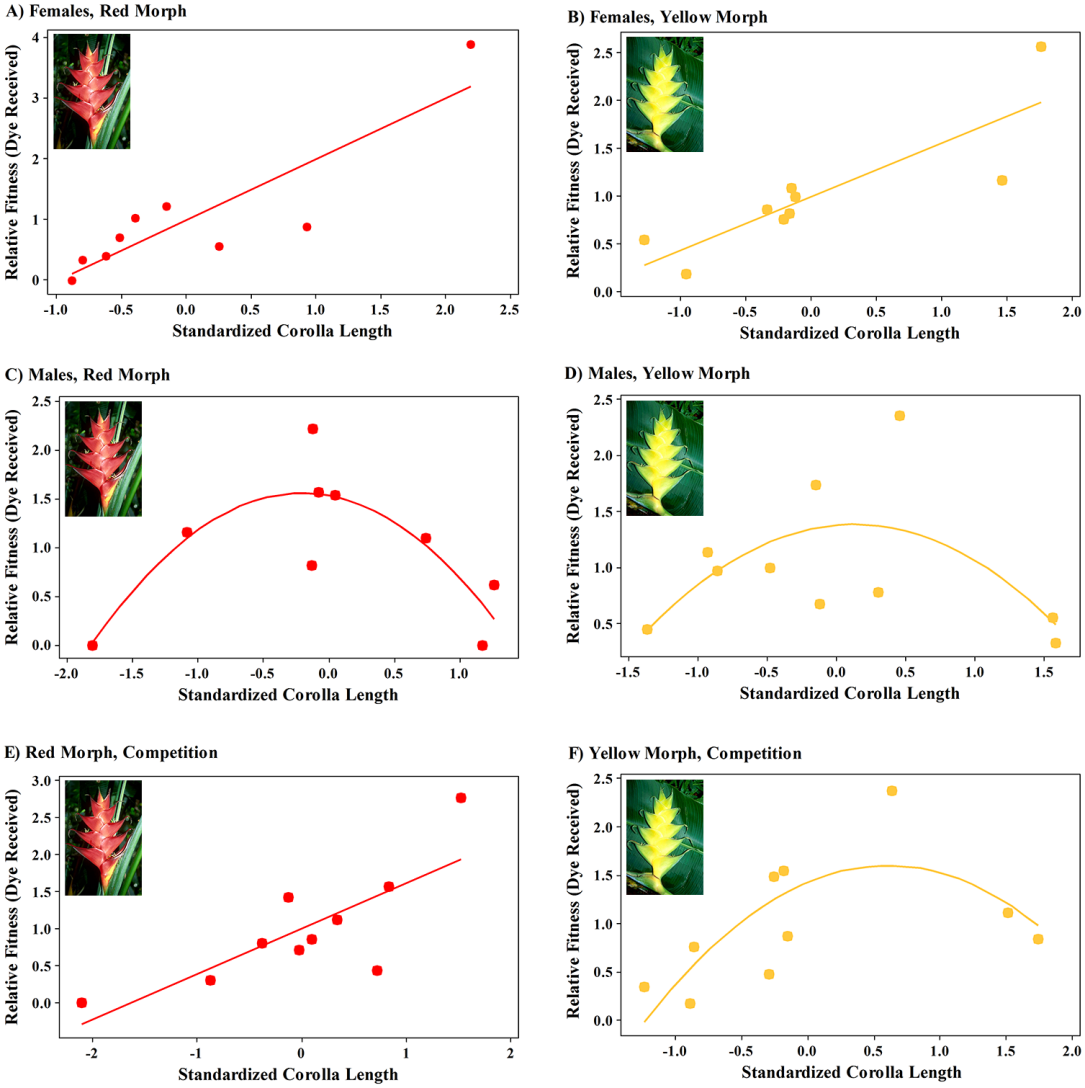
Natural selection on corolla lengths of red (A, C, E) and yellow color (B, D, F) morphs of *Heliconia caribaea* as determined from dye-pollen analogs for female (A, B) and male (C, D) purple-throated caribs when foraging alone, and for females and males in competition (E, F). A) Females, directional selection on the red morph; B) Females, directional selection on the yellow morph; C) Males, stabilizing selection on the red morph; D) Males, stabilizing selection on the yellow morph; E) Competition, directional selection on the red morph; F) Competition, stabilizing selection on the yellow morph; see Table 1 for statistics.

When foraging alone, females exerted significant directional selection for longer corollas in both the red and yellow morphs of *H*. *caribaea* (Fig 6A and 6B; Table 1; S4 Dataset). Males, on the other hand, exerted stabilizing selection on corolla lengths of both *Heliconia* morphs: for the red morph, stabilizing selection was significant and for the yellow morph, marginally so (Fig 6C and 6D; Table 1). In the competition trials, we observed significant directional selection for longer flowers of the red morph (Fig 6E; Table 1) and significant stabilizing selection for flowers of the yellow morph (Fig 6F; Table 1).

**Table 1:** Selection gradients with standard errors and sample sizes (parentheses) for corolla length and one fitness measure (dye grains per plant) for three treatments (Females, Males, Competition) and two color morphs (Red, Yellow) of *H*. *caribaea*.

Treatment, Morph	*N*	*B*_*uni*_	*R*^*2*^	*γ*_*uni*_	*R*^*2*^	*P*, *R*^*2*^
**Females, Red**	9	1.01±0.21***	0.766	0.80±0.39	0.861	0.075
**Females, Yellow**	9	0.56±0.13***	0.724	0.18±0.30	0.739	0.722
**Males, Red**	9	-0.01±0.28	0.000	-1.19±0.32**	0.700	0.006
**Males, Yellow**	10	-0.07±0.22	0.013	-0.85±0.32^†^	0.392	0.058
**Competition, Red**	10	0.61±0.17**	0.608	0.35±0.26	0.691	0.222
**Competition Yellow**	10	0.27±0.21	0.172	-0.95±0.40*	0.545	0.033

*B*_*uni*,_, univariate linear selection gradients; *γ*_*uni*_, univariate nonlinear (quadratic) selection gradients; *R*^*2*^, explanatory value of models with only linear terms and models with linear and quadratic terms, respectively; *P*, *R*^*2*^, significance of an increase in R^2^ from fitting the quadratic model.

* *P* < 0.05;

** *P* ≤ 0.01;

*** *P* < 0.005;

^†^ *P* = 0.075.

## Discussion

Our experiments provide support for predictions from foraging models of floral divergence that competition and subsequent resource partitioning by two different kinds of pollinators can increase assortative mating in floral phenotypes, leading to selection for divergence in their floral traits. Our experiments differed, however, from the structure of these models in two ways. First, in addition to assessing relative fitness of color morphs, we also measured selection on corolla length; i.e., we assessed selection on two traits, not one, as in the models. Our experiments indicate that two types of pollinators can use one floral trait (color) as a cue for resource partitioning while driving selection on a different trait (corolla length). To the extent that traits may be correlated, our results provide support for the evolution of pollination syndromes as discussed by Rodríguez-Gironés and Santamaría [10]. Second, Rodríguez-Gironés, Santamaría, Llandres, and Possingham [5, 6, 7, 8, 9, 11] conceived their models with exploitative competition in mind, whereas we conducted experiments in which pollinators interacted through interference competition. Nonetheless, Rodríguez-Gironés and Santamaría [10] noted that interference competition between pollinators also could lead to floral divergence and in theory could lead to stronger divergence if competitive exclusion was absolute. Although male purple-throated caribs did not completely exclude females from the yellow morph of *H*. *caribaea* in our experiments, the levels of assortative mating and selection for floral divergence we measured may be more extreme than what might occur in exploitative competition between pollinators if interference competition resulted in more exclusive use of floral resources than exploitative competition.

### Pollinator preference

The models of Possingham, Rodríguez-Gironés, Santamaría and Llandres [5, 6, 7, 8, 9, 11] require some asymmetry in foraging and competitive abilities between two types of pollinators for resource partitioning to occur, such as differences in visual discrimination of colors or differences in tongue length and hence nectar extraction efficiency. In our experiments, the asymmetries were male preference for the yellow morph of *H*. *caribaea* and males’ ability to dominate and exclude females through aggressive behavior. Because we controlled both the number of inflorescences and the number of flowers per plant in these experiments, these variables could not influence male preference for the yellow morph. In addition, the average nectar volume and concentration per flower and the number of bracts per inflorescence did not differ significantly between the red and yellow color morphs used in these experiments (see Methods, S1 Table). We found from surveys of *H*. *caribaea* at the Warner Road site where birds were captured that the yellow morph on average has more bracts per inflorescence, and hence more flowers, than the red morph. Thus, males' preference for the yellow morph of *H*. *caribaea* in our experiments may reflect their prior experience with these plants and their preference for the most rewarding *Heliconia* in their habitat. Such associations between color and reward have been observed in other species of nectar-feeding birds [18, 44]. In this regard, in all natural populations where we recorded significant differences in use of color morphs by male and female purple-throated caribs with males visiting the yellow morph and females visiting the red morph, the yellow morph either had significantly more bracts per inflorescence than the red morph or was under significant directional selection for more bracts per inflorescence [24, 29]. Consistent with the hypothesis that prior experience can drive preferences, in pilot experiments we conducted in 2011, three males captured from a site where only the red morph of *H*. *caribaea* is present preferred the red morph of *H*. *caribaea* to the yellow morph (unpublished data). The lack of preference exhibited by females also may result from prior experience. Females are often non-territorial and subordinate to males, and as a result may hedge their bets by visiting both color morphs of *H*. *caribaea* equally, particularly if they may be subsequently excluded from one morph by a dominant male competitor. Our competition trials demonstrate that such exclusion happens.

### Assortative mating in plants

Pollinator competition and resource partitioning increased assortative mating (dye transfer) within *H*. *caribaea* color morphs, although assortative mating was quite high for both females foraging alone, and especially for males foraging alone (Fig 4). The high assortative mating observed when males foraged alone likely resulted from their preference for the yellow morph of *H*. *caribaea* (see [45] for a similar result). In addition, both males and females repeatedly visited the same plants over the duration of our 5 h observation periods, and in many trials, some plants remained unvisited even though their flowers contained copious amounts of nectar. We observed similar patterns of plant use and revisit behavior in our field studies [26]. Assortative mating within floral phenotypes may be much lower for pollinators that do not have preferences and do not revisit plants or flowers.

### Selection on color

The red and yellow color morphs of *H*. *caribaea* received similar amounts of dye particles in the competition trials, indicating that the two morphs had equal fitnesses (Fig 5). This result, when combined with partitioning and increased assortative mating of the red and yellow colors morphs during our competition trials (Figs 3 and 4) suggests that competition between male and female purple-throated caribs would maintain both color morphs in a population. In contrast, male preference for the yellow morph and significantly greater dye deposition on the yellow morph when foraging alone (Figs 3 and 5) suggest that in a hypothetical male-only environment, male purple-throated caribs would drive the yellow morph to fixation. Assessing selection by female purple-throated caribs on *H*. *caribaea* color morphs is less clear, because females exhibited no preference for either color morph nor did they deposit significantly more dye particles on one morph as opposed to the other (Figs 3 and 5). We interpreted the lack of female preference for color morphs as a consequence of competitive exclusion by males. As noted above, however, hummingbird preferences for colors are learned behaviors [18] and as a consequence are labile. We thus might expect that in a hypothetical female-only environment, females would prefer the most rewarding or abundant *H*. *caribaea* color morph and thus drive that color morph to fixation similarly to a hypothetical male-only environment. This explanation assumes that females learn over time to associate reward with color; our pilot experiments with artificial feeders indicate that this is the case (unpublished data).

### Selection on corolla length

In our experiments, female purple-throated caribs exerted selection for longer corollas in both color morphs of *H*. *caribaea*, whereas males exerted stabilizing selection on both color morphs of *H*. *caribaea*, although for the yellow morph this selection was not quite significant (Fig 6, Table 1). As a result, in competition, resource partitioning of the red and yellow color morphs by sexes of purple-throated caribs led to directional selection for longer corollas of the red morph, and stabilizing selection on corolla length for the yellow morph (Fig 6E and 6F). Thus, under competition, divergence in corolla length was asymmetric, rather than symmetric. Our finding that males exerted stabilizing selection on corolla length is contrary to our prediction that they would select for shorter corollas in heliconias due to their shorter bills relative to females. The absence of selection for shorter corollas may be a consequence of males serving as the primary pollinator of *H*. *caribaea* [25]. As a result, corolla lengths of *H*. *caribaea* may be well-matched to the bills of males in terms of pollen deposition and receipt, leading to stabilizing selection or even no selection in populations where variation in corolla length has been greatly reduced. In contrast, longer-billed females are the primary pollinator of *H*. *bihai*, which has considerably longer flowers than *H*. *caribaea* [24, 29]. Such mismatches between bill and corolla lengths have been predicted and observed to drive directional selection on corolla length [46]. In the case of female purple-throated caribs, flowers that are short relative to their bills deposit pollen on the bills, whereas flowers that are longer relative to the bills deposit pollen on the birds’ crowns, which provide not only a greater surface area for pollen deposition but also a more favorable medium (feathers) for pollen transport [47].

Our experiments were specifically designed to create competition between males and females by providing them with limiting nectar resources. To the extent that birds used color as a cue for resource partitioning, we expected males and females to partition color morphs given that we provided them with equal numbers of the two morphs, which they did. In our studies of natural populations, we recorded significant differences in corolla length between the red and yellow morphs of *H*. *caribaea* in some populations but not in others, and measured significant selection on corolla length in some populations but not in others [24, 29]. In all populations where we recorded significant differences between color morphs in corolla length or patterns of selection, we also recorded significant differences in visit frequencies to color morphs by sexes of purple-throated caribs. Why purple-throated caribs do not partition color morphs in all populations of *H*. *caribaea* requires further study, but our experiments suggest that partitioning and divergence may depend in part on the absolute and relative abundances of color morphs, which would influence the ability of territorial males to monopolize one or both morphs. In that regard, in the two natural populations where we observed sex differences in visitation and directional selection on corolla length of *Heliconia* color morphs, ratios of morphs were 45% red and 55% yellow and 44% red and 56% yellow [29]. In contrast, in the one population where we did not observe sex differences in visitation and selection on corolla length of *Heliconia* color morphs, ratios of morphs were 74% red and 26% yellow ([29], data averaged for two years).

## Conclusions

Although our experiments were conducted in an artificial microcosm, they provide support for predictions of foraging models that pollinator competition can lead to resource partitioning, assortative mating, and selection for divergence in two floral phenotypes. Given the numerous studies that have documented competition and resource partitioning in various taxa of pollinators (see Introduction), pollinator competition should be viewed as one of many mechanisms for floral divergence. Pollinator competition as a mechanism for floral divergence would seem especially likely in the diversification of bird-pollinated plants, given that numerous avian nectarivores are territorial, especially if competitive exclusion through territoriality is absolute [10].

Our experiments also provide a novel perspective for the diversity of floral colors in plants. Research over the past 20 years indicates that variation in floral color within and between plant species may result from pollinator preference, pollinator avoidance, pollinator constancy, competition between plant species, herbivory, environmental effects, and genetic drift [48, 49, 50, 51, 52, 53]. The experiments presented here, as well as our field observations [23, 24, 29], suggest that pollinator competition should be viewed as an additional mechanism that can drive, or at least maintain, different floral color phenotypes within a population.

## Supporting Information

S1 Dataset Dye particles and pollen grains received versus flower order. (XLS)

S2 Dataset Proportions (untransformed) of male and female visits to the yellow morph of *H*. *caribaea* alone and in competition. (XLS)

S3 Dataset Proportions (untransformed) of dye transfers within *Heliconia* morphs for males alone, females alone, and males and females in competition. (XLS)

S4 Dataset Mean corolla lengths and mean numbers of dye particles received per plant for analyses of selection on corolla length for males alone, females alone, and males and females in competition. Prior to analysis we standardized corolla length to a mean equal to zero and to a variance equal to one and converted our fitness measure, mean dye particles received per plant, to relative fitness by dividing individual fitness by mean fitness.
(XLS)

S1 Fig Number (Mean ± SE) of dye (open circles) and pollen grains (solid circles) deposited versus flower presentation order. (TIF)

S1 Table Statistical comparison of nectar volume, nectar concentration, number of bracts per inflorescence, and corolla length between plants of the red and yellow morphs of *H*. *caribaea* used in our experiments in 2012, 2013, and 2015. (DOC)

S1 Text Suitability of powdered dyes as a proxy for pollen grains in *Heliconia caribaea*. We provide detailed statistics comparing (i) the slopes and intercepts of the regressions of dye particles/pollen grains received versus flower order and (ii) the number of dye particles or pollen grains received for flowers of a given order.
(DOC)
