# Supplementary material for: Pollinator Competition as a Driver of Floral Divergence: An Experimental Test
Source: PLoS One. 2016 Jan 27;11(1):e0146431. doi: 10.1371/journal.pone.0146431 (PMC4729399; doi:10.1371/journal.pone.0146431)
Supplement: S1 Table — (DOC) [file pone.0146431.s006.doc]

**S1 Table. Statistical comparison of nectar volume (µl), nectar concentration (% sucrose), number of bracts per inflorescence, and corolla length (mm) between plants of the red and yellow morphs of *H. caribaea* used in our experiments in 2012 (7 red, 7 yellow; 65 flowers), 2013 (7 red, 9 yellow; 88 flowers), and 2015 (8 red, 9 yellow; 74 flowers). Nectar was sampled in late afternoon (approximately 1500 h).**

| **Plant Trait** | **Red Morph**  **(Mean ± SE)** | **Yellow Morph**  **(Mean ± SE)** | ***t*** | ***P*** |
| --- | --- | --- | --- | --- |
| **Nectar Volume 2012** | 92.1 ± 11 | 98.8 ± 13 | 0.40 | 0.70 |
| **Nectar Concentration 2012** | 21.3 ± 0.8 | 21.0 ± 1.7 | 0.21 | 0.84 |
| **Bracts per Inflorescence 2012** | 9.44 ± 0.67 | 8.55 ± 0.58 | 1.01 | 0.33 |
| **Corolla Length 2012** | 34.51 ± 0.19 | 35.27 ± 0.43 | 1.62 | 0.14 |
| **Nectar Volume 2013** | 98.3 ± 6.5 | 105.1 ± 8.5 | 0.64 | 0.53 |
| **Nectar Concentration 2013** | 21.8 ± 0.8 | 22.4 ± 1.7 | 0.55 | 0.59 |
| **Bracts per Inflorescence 2013** | 8.27 ± 0.44 | 7.59 ± 0.32 | 1.26 | 0.23 |
| **Corolla Length 2013** | 34.52 ± 0.27 | 35.53 ± 0.52 | 1.71 | 0.12 |
| **Nectar Volume 2015** | 119.8 ± 9.3 | 121.9 ± 5.6 | 0.19 | 0.85 |
| **Nectar Concentration 2015** | 22.9 ± 0.7 | 22.8 ± 0.6 | 0.09 | 0.93 |
| **Bracts per Inflorescence 2015** | 6.56 ± 0.33 | 6.79 ± 0.23 | 0.56 | 0.59 |
| **Corolla Length 2015** | 34.55 ± 0.13 | 34.65 ± 1.25 | 0.19 | 0.85 |
