# Supplementary material for: Pollinator Competition as a Driver of Floral Divergence: An Experimental Test
Source: PLoS One. 2016 Jan 27;11(1):e0146431. doi: 10.1371/journal.pone.0146431 (PMC4729399; doi:10.1371/journal.pone.0146431)
Supplement: S1 Text — We provide detailed statistics comparing (i) the slopes and intercepts of the regressions of dye particles/pollen grains received versus flower order and (ii) the number of dye particles or pollen grains received for flowers of a given order. (DOC) [file pone.0146431.s007.doc]

**S1 Text. Suitability of powdered dyes as a proxy for pollen grains in *Heliconia caribaea***. To verify that fluorescent powdered dye was an appropriate analog for pollen, we conducted pollen carryover trials using 10 purple-throated caribs, Each of the 10 birds was offered a newly-dehisced *H. caribaea* flower to which dye had been liberally applied with a toothpick, and then was allowed to visit four to six emasculated flowers in succession. The number of dye particles received per flower was a significant predictor of the number of pollen grains received per flower (Pollen = 0.44 + 0.88Dye; *r^2^* = 0.9, *F* = 502.5, *P* < 0.001, *N* = 54). The slope and intercept of the regression of the number of dye particles received versus flower order (Slope ± SE: -2.38 ± 0.20; Intercept ± SE: 12.1 ± 0.72) did not differ significantly from the slope and intercept of the number of pollen grains received versus flower order (Slope ± SE: -2.19 ± 0.19; Intercept: 11.4 ± 0.67; Analysis of covariance test of slopes: *F* = 0.45, *P* = 0.50; Analysis of covariance test of intercepts: *F* = 0.05, *P* = 0.83). Similarly, the number of dye particles or pollen grains received for flowers of a given order did not differ significantly for the first (Mean ± SE, Dye: 11.4 ± 1.0; Pollen: 10.8 ± 0.8; *t* = 0.85, *P* = 0.41), second (Mean ± SE, Dye: 6.6 ± 0.7; Pollen: 6.3 ± 0.8; *t* = 0.76, *P* = 0.45), third (Mean ± SE, Dye: 3.7 ± 0.6; Pollen: 3.4 ± 0.5; *t* = 0.71, *P* = 0.49), fourth ((Mean ± SE, Dye: 2.0 ± 0.60; Pollen: 2.5 ± 0.6; *t* = -1.63, *P* = 0.14), fifth (Mean ± SE, Dye: 0.22 ± 0.22; Pollen: 0.44 ± 0.34; *t* = -0.61, *P* = 0.56) or sixth (Mean ± SE, Dye: 0.00 ± 0.00; Pollen: 0.00 ± 0.00; no statistics since all values were zero) in the sequence (see S1 Fig). Thus, powdered dyes are a good proxy for pollen grains in *H. caribaea*.
